# Development of Droplet Digital PCR-Based Assays to Quantify HIV Proviral and Integrated DNA in Brain Tissues from Viremic Individuals with Encephalitis and Virally Suppressed Aviremic Individuals

**DOI:** 10.1128/spectrum.00853-21

**Published:** 2022-01-12

**Authors:** Hye Kyung Chung, Julian B. Hattler, Jigna Narola, Harita Babbar, Yanhui Cai, Mohamed Abdel-Mohsen, Woong-Ki Kim

**Affiliations:** a Advanced BioScience Laboratories, Inc., Rockville, Maryland, USA; b Department of Microbiology and Molecular Cell Biology, Eastern Virginia Medical School, Norfolk, Virginia, USA; c The Wistar Institute, Philadelphia, Pennsylvania, USA; Memorial Sloan Kettering Cancer Center

**Keywords:** antiretroviral therapy, brain, droplet digital PCR, HIV-1, HIV-associated neurocognitive disorders, reservoir

## Abstract

Although combination antiretroviral therapy (cART) can suppress the replication of HIV, the virus persists and rebounds when treatment is stopped. To find a cure that can eradicate latent reservoir, a method should be able to quantify the lingering HIV. Unlike other digital PCR technologies, droplet digital PCR (ddPCR), provides absolute quantification of target DNA molecules using fluorescent dually labeled probes by massively partitioning the sample into droplets. ddPCR enables exquisitely sensitive detection and quantification of viral DNA from very limiting clinical samples, including brain tissues. We developed and optimized duplex ddPCR assays for the detection and quantification of HIV proviral DNA and integrated DNA in the brain of HIV-1-infected patients. We have applied these approaches to successfully analyze 77 human brain tissues obtained from 27 HIV-1-infected individuals, either fully virally suppressed or with encephalitis, and were able to quantify low levels of viral DNA. Further developments and advancement of digital PCR technology is promising to aid in accurate quantification and characterization of the persistent HIV reservoir.

**IMPORTANCE** We developed ddPCR assays to quantitatively measure HIV DNA and used this ddPCR assays to detect and quantitatively measure HIV DNA in the archived brain tissues from HIV patients. The tissue viral loads assessed by ddPCR was highly correlative with those assessed by qPCR. HIV DNA in the brain was detected more frequently by ddPCR than by qPCR. ddPCR also showed higher sensitivity than qPCR since ddPCR detected HIV DNA signals in some tissues from virally suppressed individuals while qPCR could not.

## INTRODUCTION

Despite effective combination antiretroviral therapy (cART), HIV survives in an integrated form and almost always rebounds when treatment is interrupted (1–3). While most of the integrated proviruses in these reservoirs are defective, a small fraction of those that are genetically intact and replication-competent persists during cART (4–6). The proviral landscape during cART remains poorly characterized and accurately measuring the reservoir of persistent HIV proviruses is critical to assessing the effectiveness of curative strategies aimed at HIV eradication. As has been previously pointed out by many, a universal, precise, reliable, and high-throughput assay to measure the amount of infectious virus remaining in the body or to confirm the complete elimination of the latent reservoir *in vivo* would be ideal, and a key step on the path to HIV eradication (7). In practice, no assay quantifies the HIV reservoir accurately (8), and it is likely that input from a variety of assays will be necessary to understand the size and complexity of HIV reservoirs.

Many assays have been developed for measuring HIV infection utilizing amplification of nucleic acid sequences by quantitative PCR (qPCR), which has been used extensively to diagnose HIV-1 infection and monitor antiretroviral regimens (9). Several qPCR assays have been developed that detect HIV-1 proviral DNA, RNA load, integration by Alu-PCR, and the long terminal repeat (LTR) region (10–13). While these methods have been widely used in virology, qPCR requires a prevalidated standard curve or endogenous controls to estimate concentration in unknown samples. Inefficient amplification in the standard curve limits the accuracy of qPCR. Target sequence variation, as well as instrument and operator variability, are also limitations of this method.

Quantification of integrated HIV DNA has been a challenge. For example, lower molecular-weight HIV 1-LTR and 2-LTR circles that have the same sequences as integrated HIV provirus accumulate in the nuclei of infected cells in the event of failed HIV DNA integrations. Attempts to measure integrated HIV DNA have involved utilizing primer/probe sets which detect host cell Alu sequences in addition to HIV sequences. The variable distances of Alu sequences from each proviral sequence range up to thousands of base pairs, and they have required correction factors, owing to varying amplification efficiencies, to calculate proviral DNA. For these reasons, Alu-gag qPCR has proven to be a technically complex assay. To address the challenge of distinguishing proviral DNA from lower-molecular weight species of HIV DNA, De Spiegelaere et al. investigated a strategy to subtract a background (from PCR of the low molecular weight HIV DNA) from Alu-HIV PCR (14).

Droplet digital PCR (ddPCR) is an endpoint PCR technique that uses sample partitioning to obtain absolute quantification without the need for a standard curve (15). Amplification of target sequences occurs in 20,000-nanoliter-sized oil droplets, in 20 μL of reaction volume, to estimate an absolute count of target DNA. The massive sample partitioning creates a dynamic range from a single copy up to 100,000 copies, with Poisson corrections extending the range to multiple copies per droplet (16). Numerous studies published to date have measured HIV-1 DNA, RNA, and 2-LTR with ddPCR (17–21); several have directly compared ddPCR to qPCR, the gold standard methodology for quantification of HIV-1 DNA and integrated DNA, as reviewed by Trypsteen et al. (22). Overall, ddPCR has shown to be superior in accuracy, precision, and reproducibility compared to qPCR, but it is not always more sensitive (in measuring the smallest concentration of an analyte). ddPCR is also more resilient to mismatches between the primers/probes and the target sequence, which are often observed in HIV quantification.

Here, we developed and tested two ddPCR methods to measure levels of HIV DNA in brain tissues: (i) total HIV DNA with RPP30 as an internal control, and (ii) integrated HIV DNA following a nested-ddPCR performed using gag-reverse HIV primer and Alu-forward primer. Both assays were developed and optimized with the Bio-Rad ddPCR system. We then applied these ddPCR methods to quantitate total HIV and integrated DNA in 27 HIV-infected individuals with or without cART treatment. A total of 77 tissues from three anatomical brain sites were tested. The results from these studies provide key insights into residual HIV infection in tissues following cART treatment.

## RESULTS

### Optimization of the annealing temperature with duplex droplet digital PCR assays.

**FIG 1:**
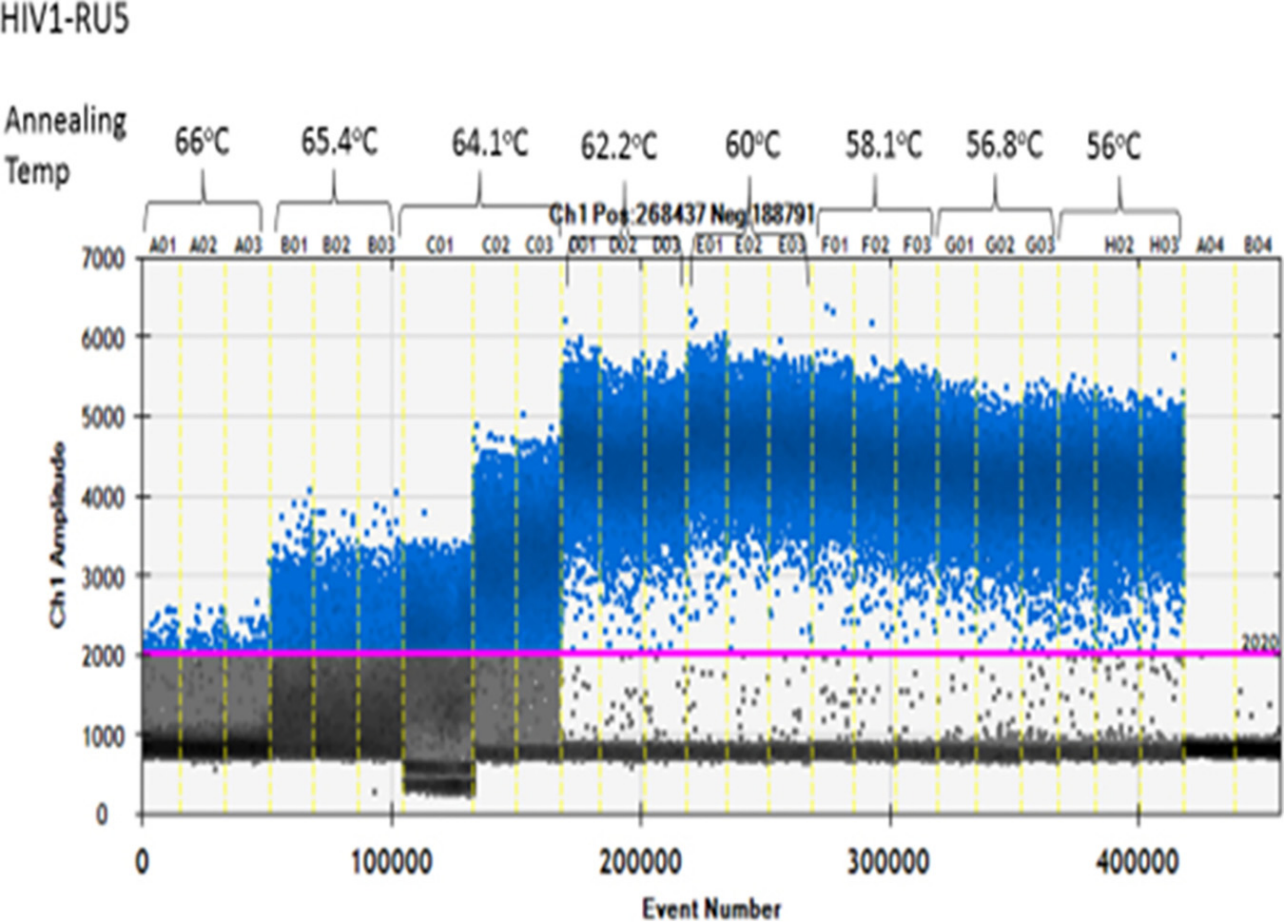
Evaluation of HIV DNA assay conditions. HIV-1 primers and probes, at final concentrations of 500 nM and 250 nM, respectively, were tested in PCR. To evaluate optimal PCR conditions, the primers and probe at those concentrations were tested under an annealing temperature gradient of 56°C to 66°C.

Optimization of annealing temperature was critical for reaction specificity. As shown in Fig. 1, primers and probes at final concentrations of 250 nM and 500 nM, respectively, were optimal, with an annealing temperature of 58.1°C (Fig. 1 and Table 1). To select an annealing temperature, we chose thresholds that separated positive and negative droplets in both channels.

**TABLE 1:** Annealing temperature gradient

Mean HIV copies per 20 μL	Annealing temp (°C)
1.23E + 02	66.0
1.93E + 03	65.4
3.21E + 04	64.1
5.33E + 04	62.2
5.32E + 04	60.0
5.35E + 04	58.1
5.17E + 04	56.8
5.30E + 04	56.0

To evaluate the optimal concentration of primers for integrated DNA assay, we tested Alu-forward and HIV-1 Gag-reverse primers with final concentrations of 200 nM and 1200 nM, respectively, in PCR. Next, to establish optimal PCR conditions, we performed an annealing temperature gradient PCR, as described below. The temperature gradient was between 50°C and 60°C. We selected 60°C as the annealing temperature for the second PCR of integrated HIV-1 DNA nested ddPCR (data not shown).

### HIV DNA quantification with ddPCR assay.

**FIG 2:**
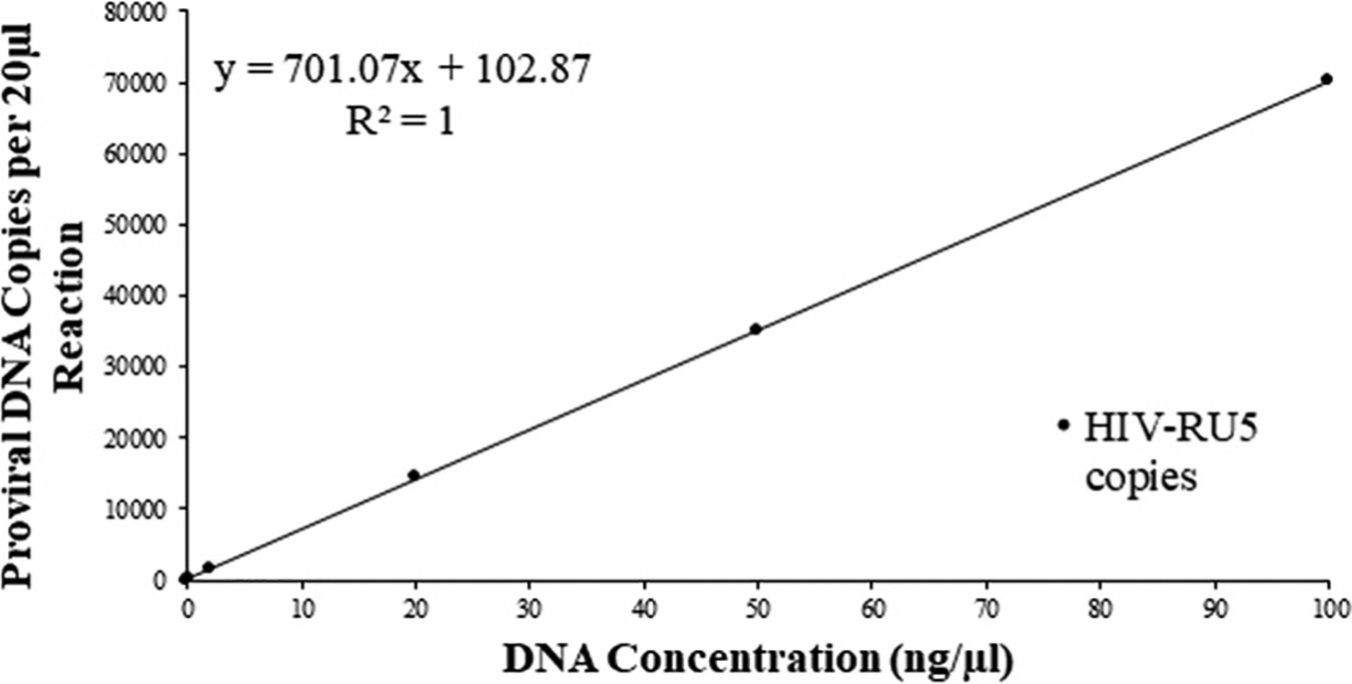
The limit of quantitation (LOQ) was determined for the ddPCR assay by diluting the DNA. Three replications of various concentrations were then evaluated with ddPCR to determine the lowest copy number which exhibited amplification in all reactions at that concentration. The RPP30 was also included in the samples to show that there were no inhibitions present in the reaction that could have caused false negatives. The LOD was the lowest dilution that had 100% amplification. As shown in Table 1, the data are very consistent for each concentration between 0.2 ng/μL and 100 ng/μL. A minimum DNA concentration of 0.2 ng/μL can be used for the quantitation of proviral DNA by ddPCR assay.

To assess the dynamic detection range of HIV DNA, the limit of quantitation (LoQ) was determined with a ddPCR assay by diluting the DNA extracted from Molt3-IIIB cell line. Three replications of various concentrations were then evaluated with ddPCR to determine the lowest copy number which exhibited amplification in all reactions at that concentration. The RPP30 was also included in the samples to show that there were no inhibitions present in the reaction that could have caused a false negative. The LoQ was the lowest dilution that had 100% amplification. As shown in Table 2 and Fig. 2, quantification of HIV proviral DNA by HIV-1-RU5 primer set was very consistent, with ∼1.46 copy/cell regardless of input DNA concentration range (0.2 to 100 ng/μL). A linear correlation was observed and confirmed the quantitative accuracy of the limit dilution method at higher amounts of HIV DNA. Even though the sensitivity of this assay is ∼1 copy/μL of reaction at an input DNA concentration of 0.02 ng/μL, a minimum concentration of 0.2 ng/μL of DNA can be used for accurate quantitation of HIV DNA in patient samples by optimized ddPCR assay. Our data from different DNA concentrations, ranging from 0.5 to 5 ng/μL, suggested that a concentration of 5 ng/μL was optimal for accuracy and consistency.

**TABLE 2:** Selection of input DNA concentration

Concn. of HIV DNA samples (ng/μL)	Mean HIV copies per 20 μL	HIV copies per 10^6^ cells
0.02	2.60E + 01	2.11E + 06
0.2	1.56E + 02	1.45E + 06
2	2.79E + 03	1.49E + 06
20	2.14E + 04	1.45E + 06
50	5.36E + 04	1.47E + 06
100	7.45E + 04	1.47E + 06

### HIV-1-infected human brain proviral and integrated DNA detection.

**FIG 3:**
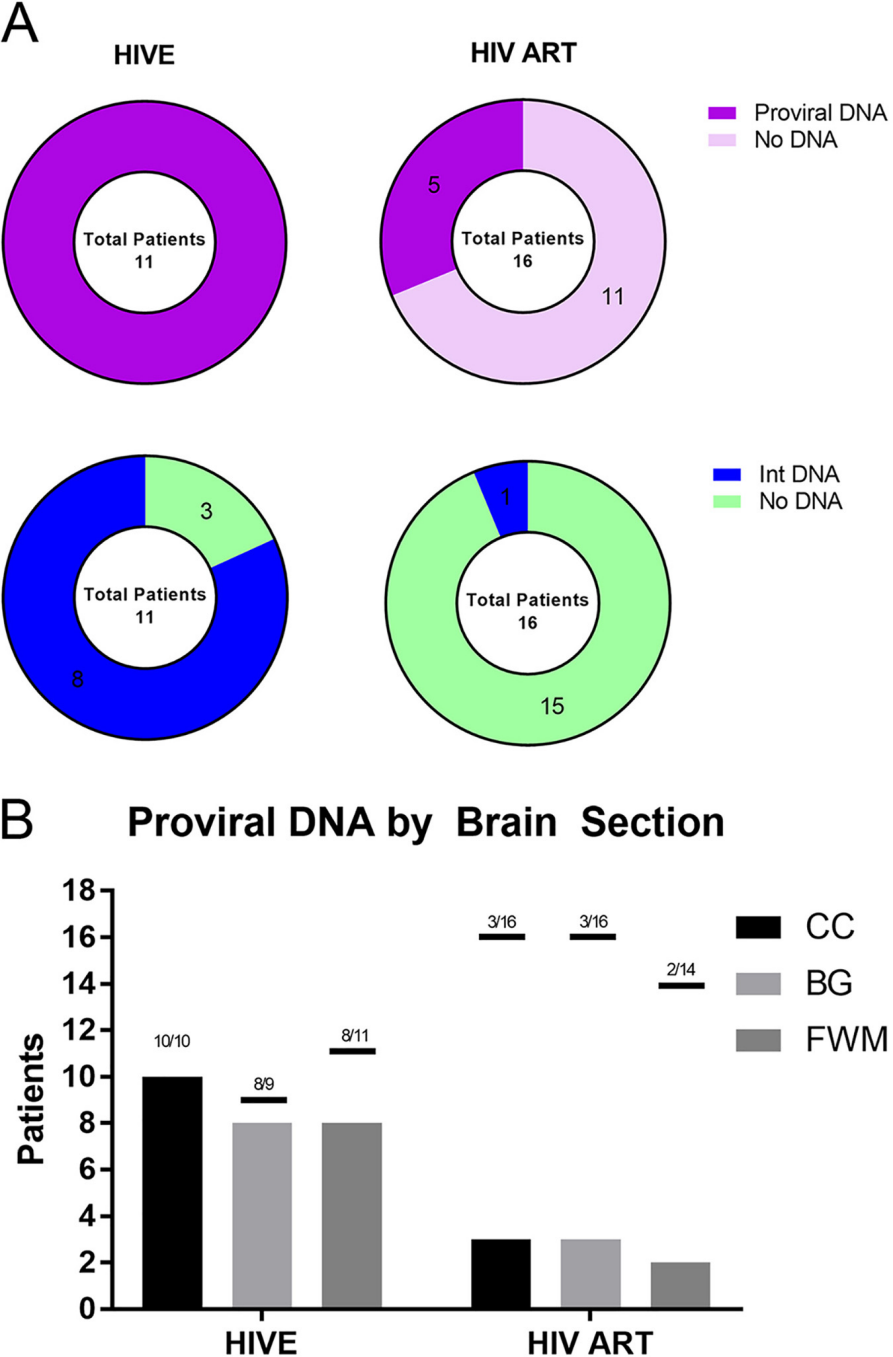
Total HIV-1 DNA and integrated DNA by droplet digital PCR for HIVE and HIV cART samples. HIVE and HIV cART patients testing positive for proviral DNA (purple) and integrated DNA (blue) (A). Proviral DNA in brain sections were tested by ddPCR. Corpus callosum (CC), basal ganglia (BG), and frontal white matter (FWM) from each patient, showing proviral DNA (B). Not all patients had all three sections available.

Following optimization of the ddPCR assay, we attempted to detect proviral and integrated DNA from our cohort of 27 HIV-1-infected individuals, as well as from the 2 HIV-negative controls. Out of the 77 sections tested, 40 were found to have proviral DNA ranging from 10° to 10^6^ HIV-1 DNA copies/10^6^ cells, with 19 also harboring HIV-1 integrated DNA ranging from 10^3^ to 10^4^ integrated DNA copies/10^6^ cells. Of note, DNA was detected at levels as low as 4 copies/10^6^ cells. Unsurprisingly, proviral DNA was detected in all patients with HIV-1 encephalitis (HIVE), with most patients harboring DNA in all brain sections tested (8/11 patients), with the exception of sections 00039 FWM, 00284 BG and FWM, and 01212 FWM. Despite below-detection plasma viral loads, proviral DNA was also detected in samples from virally suppressed individuals (10/16 patients) (Fig. 3A and B). While the majority of integrated DNA was detected in HIVE samples, integrated viral DNA was also detected in one virally suppressed individual. Integrated DNA was detected in almost all HIVE patients (9/11); however, even with very high titers of proviral DNA in sections 01555 BG, FWM, CC, and 00488 FWM, we were unable to quantify integrated DNA. There were no statistical differences in detection rates between the CC, BG, and FWM brain sections (chi-squared test).

### Comparison with qPCR.

**FIG 4:**
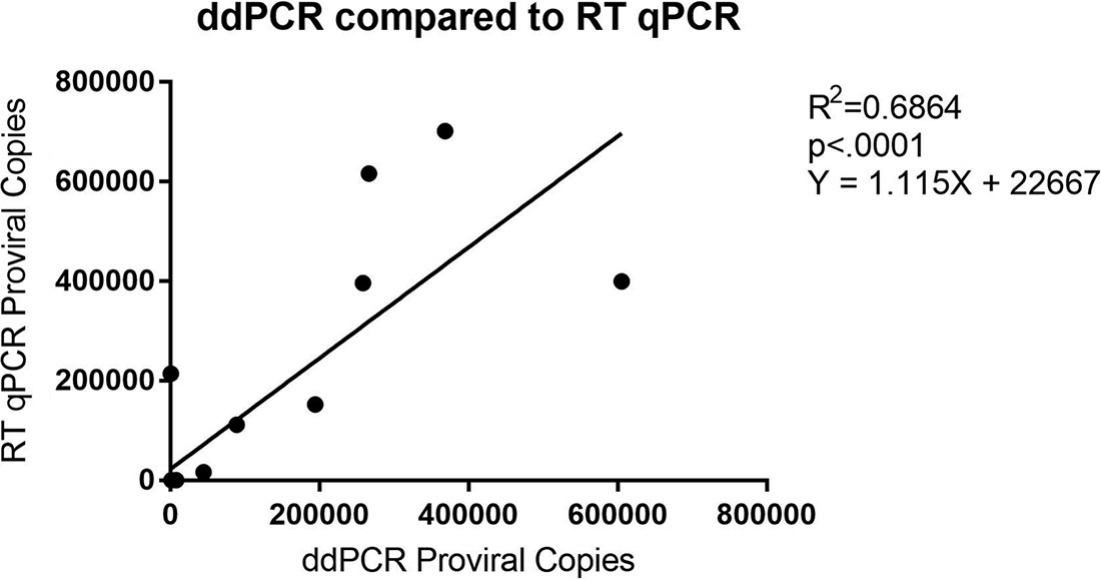
Pearson’s correlation and linear regression of 25 samples measured with both ddPCR and RT-qPCR for proviral copy number. R^2^ = 0.6864 and *P* value < 0.0001.

To further validate the ddPCR assay, proviral DNA detection was also attempted through RT-qPCR on 26 of the original 77 samples. Of these 26 samples, ddPCR detected DNA in 16 samples and qPCR detected DNA in 10 samples. A linear regression was performed and, following the removal of a single outlier, had R^2^ and *P* values of 0.6864 and <0.0001, respectively, and a slope of 1.115, showing comparable accuracy with current standard methods (Fig. 4). Of note, while the two methods provided comparable results at higher copy numbers, ddPCR detected virus at low levels (<10^4^ HIV-1 DNA/10^6^ cells) where qPCR detected no virus in eight cases, and qPCR only detected virus where ddPCR did not in two brain regions in one case (00284); taken together, these results indicate an increased sensitivity of ddPCR in this experimental setting. Additionally, of the 13 virally suppressed brain sections evaluated by both methods, ddPCR detected virus in six while qPCR did not detect proviral DNA in any.

## DISCUSSION

Despite fully suppressive cART, the proviral latency of HIV-1 remains a principal obstacle to curing the infection (23). Therefore, there is an urgent need for laboratory assays to quantify the exceptionally low levels of replication-competent viral reservoirs post-cART. ddPCR quantitation of HIV-1 DNA in brain tissues perhaps offers the best chance for a scalable, high-throughput assay for the latent reservoir. Here, we developed and optimized droplet digital assays for quantitating persistent HIV-1 DNA and integrated DNA in patients. This involved two analytical approaches and three different sections of brain tissue samples. Assays were carried out using well-characterized brain tissues from patients who were either on long-term stable cART or were not on cART and had HIVE. First, we evaluated duplex ddPCR assays for HIV-1 DNA. Using our novel ddPCR assay, we detected HIV-1 DNA in the majority of the BG, FWM, and CC sections of brain tissues from 11/11 HIVE subjects, while we detected integrated DNA in 9/11 HIVE subjects by using nested ddPCR with an integration-rate percentage ranging from 1.23% to 1.46%.

Digital droplet PCR is the division of a sample into 20,000 replicate-endpoint PCRs that are termed either positive or negative. These binary data are processed with Poisson statistics to obtain absolute quantification (14). Direct absolute quantification of endpoint PCR increases quantitative accuracy, alleviates the need for a standard curve, and increases flexibility in assay design (16). Most current digital PCR platforms provide the ability to make high numbers of technical replicates from a single sample. The recently developed ddPCR assay has a 1 copy/μL to 10^5^ copies/μL dynamic range and the potential to be more accurate, precise, and sensitive than real-time qPCR assay. Real-time qPCR is a current gold standard for HIV-1 DNA and integrated DNA quantitative methods and is widely used in the field. To confirm our observation, we measured HIV-1 DNA in selected HIVE and virally suppressed brain tissues samples using real-time qPCR. In comparing the results, the tests showed similar accuracy (Fig. 4); however, ddPCR was able to detect low levels of virus where qPCR could not. To date, ddPCR has been shown to produce greater accuracy, precision, and reproducibility while being unable to match qPCR in sensitivity. Here, we show not only the accuracy of our ddPCR assay, but also its increased sensitivity compared to real-time qPCR.

cART has become the standard treatment for HIV and AIDS, both extending life and drastically increasing quality of life for patients. However, cART is not a cure, and many patients suffer from HIV-associated neurocognitive disorders (HAND) while receiving treatment, with the cessation of treatment leading to viral rebound from viral reservoirs. To truly cure HIV, this reservoir must be found, accurately measured, and then eradicated. Yet to date, no assay can accurately measure this reservoir, with the field currently relying heavily upon qPCR, which is seen as the gold standard despite acknowledgement of its inadequate sensitivity. We examined 16 aviremic, virally suppressed patients by ddPCR, quantifying viral DNA in 10 of them. Of the 16, 12 were also examined by qPCR, including 6 who were found to have proviral DNA. qPCR detected zero proviral DNA in all virally suppressed samples tested. Recent work published out of our lab (24) found viral DNA and RNA in perivascular macrophages in the brain of virally suppressed patients through *in situ* hybridization, consistent with our results, showing the brain as a viral reservoir. With ddPCR’s increased ability to detect viral reservoir in the brain demonstrated, other tissues that are suspected of harboring replication-competent virus, such as lymphoid tissue, should be tested, both to determine reservoir sites and to delineate major and minor contributions to the whole-body reservoir.

We observed the highest proviral DNA in some of the HIVE patients’ samples without integrated DNA detection. For instance, patient 01555 was determined to have a high amount of proviral DNA by ddPCR in all three brain tissues, BG, FWM, and CC, but no detectable integrated DNA were measured. Theoretically, when high virus replication occurs, there are unintegrated DNA in abundance, such as 2-LTR circles, which provide sites for PCR amplification, confound integrated HIV DNA measurements (25), and gag products in the cells, which can cause inaccuracy in PCR measurements. We speculated that the samples might contain unusually high 2-LTR circles that prevented the measurement of the integrated DNA in the developed ddPCR assay. To answer this question, we measured 2-LTR circles by real-time qPCR to interpret our results. Although total viral DNA copy numbers were comparable using both ddPCR and real-time qPCR assays, 2-LTR circles copy numbers were unexpectedly low and had no apparent relationship with integrated DNA. Thus, we ruled out the possibility of unintegrated DNA interference with the accurate measurement of integrated DNA. The second possibility is that due to a high gag product background in active replication, integrated DNA was not accurately calculated by the nested-ddPCR method that we developed. To overcome this potential false negative, patient samples with extremely high amounts of HIV DNA were run with repeated dilutions to minimize gag DNA. We were indeed able to overcome the initial false-negative integrated HIV DNA results from tissue samples with extremely high levels of HIV proviral DNA (MHBB519 CC and MHBB537 BG) by decreasing the amount of input DNA from 5 ng/μL to as little as 0.5 ng/μL. Although the lowest dilution of 0.5 ng/μL DNA sample revealed accurate and consistent results in our experiments on tissue samples with extremely high levels of HIV proviral DNA, further dilutions may be necessary to measure levels of HIV integrated DNA from samples with even higher levels of HIV proviral DNA.

In conclusion, combining two developed ddPCR and nested-ddPCR assays for HIV nucleic acid detection with the Bio-Rad ddPCR platform can enable significantly improved nucleic acid detection sensitivity in brain tissues, which is of specific interest for aviremic cART cases. These assays offer potential valuable tools for evaluating treatment strategies aimed at reducing the latent reservoir and curing viral infection.

## MATERIALS AND METHODS

### HIV-infected cell line.

HIV-IIIB infected cell line Molt-IIIB was previously made by infecting Molt cells with HIV-IIIB virus, kindly provided by Ranajit Pal. As a negative control, peripheral blood mononuclear cells (PBMCs) were isolated from human buffy coat by density gradient centrifugation and frozen to −80°C at a cooling rate of 1°C/min in 90% fetal calf serum with 10% dimethyl sulfoxide. Samples were stored at −150°C until further analysis.

### Human brain tissues.

Brain tissue for this study was provided by the National NeuroAIDS Tissue Consortium (NNTC). Autopsied brain specimens were chosen from 2 groups: untreated HIV-1 infected patients with encephalitis (HIVE), and HIV-1-infected, virally suppressed individuals. Virally suppressed cases with encephalitis, hemorrhage dura/leptomeninges, and bacterial parenchymal infection were excluded. Age and sex matching was performed to the best of our ability based on specimen availability. Fresh frozen frontal cortex (FC), corpus callosum (CC), and basal ganglia (BG) from 27 patients were successfully analyzed for a total of 75 samples, not all of these three sections were available from some individuals. All postmortem intervals were under 48 h.

### DNA isolation from Molt3-IIIB cell line.

We isolated DNA from Molt3-IIIB cell line and PBMCs. Cells were pelleted by 1-min centrifugation at full speed after thawing. After removing supernatant, cells were washed twice with 1 mL 1× phosphate-buffered saline. For DNA isolation, we used the DNeasy blood and tissue kit (Qiagen, Germantown, MD) following the manufacturer’s suggested protocol. Briefly, 360 μL Buffer ATL was added to the cell pellet followed by 40 μL Proteinase K, mixing by vortex, and incubating at 56°C for 30 to 60 min to lyse the cells. After incubation, 400 μL Buffer AL and 400 μL 96 to 100% ethanol were added, respectively, and the solution was mixed thoroughly after each addition step. This mixture was then added to a DNase spin column (600 μL). The columns were centrifuged at 8,000 rpm for 1 min. For wash steps, 500 μL AW1 buffer and 500 μL AW2 buffer were used, respectively, and columns were centrifuged at 8,000 rpm for 1 min. To ensure residual removal after AW2 buffer wash, columns were centrifuged for 2 min at 14,000 rpm. DNA was eluted by adding 100 μL AE buffer, incubating for 2 min at room temperature, and centrifuging for 3 min at 8,000 rpm. The elution step was repeated by adding 100 μL AE buffer to ensure that all DNA was eluted from the column. DNA was further concentrated by adding a 1/10 volume of 3 M NaOAc (pH 5.2) and 2.5 volumes of 100% ethanol to each sample and incubating at −20°C overnight. After overnight incubation, the samples were centrifuged at 12,000 rpm for 20 min at 4°C to pellet down the DNA. After very carefully discarding the supernatant, 500 μL of chilled 70% ethanol was added, mixed, and centrifuged at 9,000 rpm for 5 min at 4°C. Supernatant was removed carefully and completely, and the pellet was air dried for 20 min in a biosafety cabinet to ensure the removal of ethanol residue. The pellet was resuspended in 10 mM Tris and, to ensure homogeneity, samples were warmed at 63°C for 10 min. The DNA was quantified by OD_260_ measurements with a NanoDrop ND2000 Spectrophotometer.

### Genomic DNA isolation from brain tissue sections.

For DNA isolation, we used the DNeasy blood and tissue kit (Qiagen) following the manufacturer’s instructions. Briefly, 360 μL Buffer ATL was added to 25 mg of tissue, followed by 40 μL Proteinase K, mixing by vortex, and incubating at 56°C for 1 to 2 h to lyse the tissue. After homogenizing the tissue, we followed the same procedure as described in the previous section.

### Droplet digital PCR workflow.

**FIG 5:**
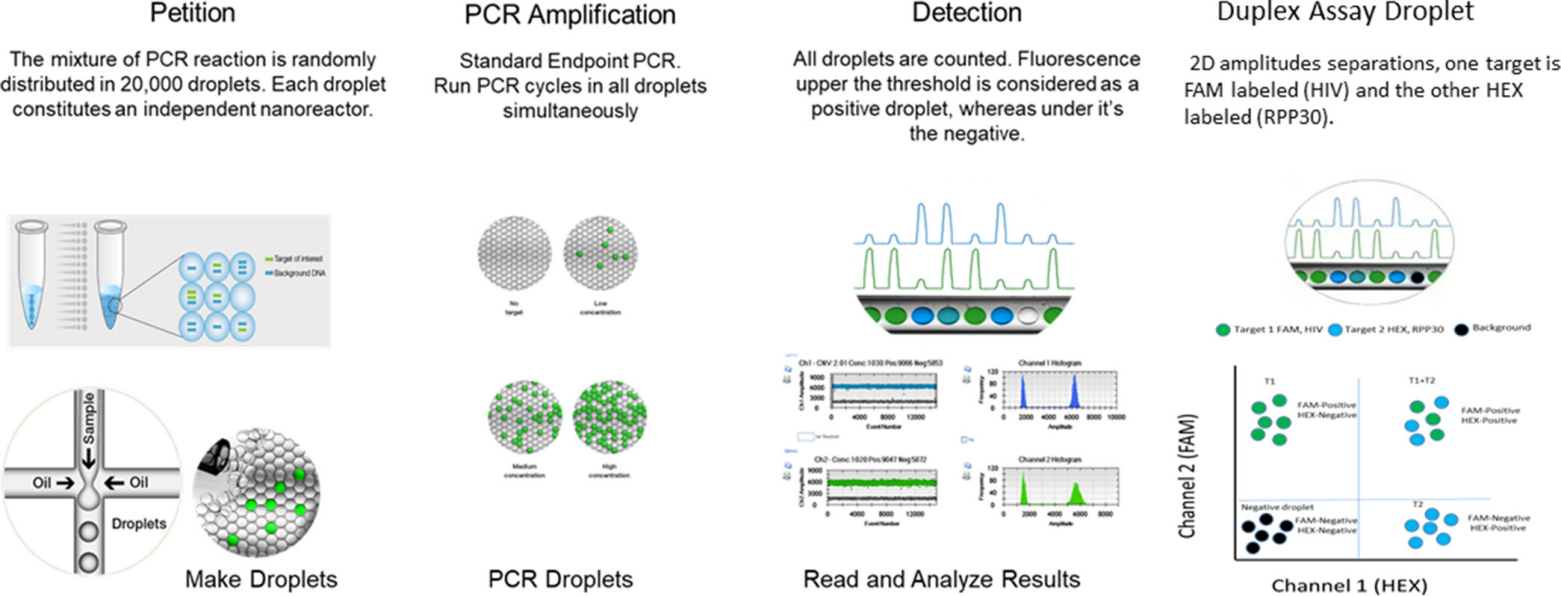
Visualized workflow of ddPCR assay. QX200 Droplet Digital PCR System (Bio-Rad) consists of automated droplet generator, thermal cycler, and droplet reader. Positive droplets (blue or green) and negative droplets (gray) can be finally analyzed with the QuantaSoft software, where thresholds should be set between the negative and positive droplets.

A Bio-Rad QX200 ddPCR system with an automatic droplet generator, as described in Fig. 5, was used for this study. Briefly, instead of one tube of standard PCR, the reaction mixture was partitioned randomly and distributed in 20,000 droplets. Each droplet contains target sequence, primers, dual quenched probes, and PCR master mix with enzyme. The PCR amplification occurred in all droplets simultaneously with similar amplification efficiencies. For limit of blank (LoB), 6 HIV-negative human PBMC samples (negative control) and 6 no-template controls (NTC) were used. The HIV-infected Molts-IIIB cell line was used as a positive control. To determine the limit of detection (LoD), a total of eighteen 10-fold or 2-fold serial dilutions of Molts-IIIB DNA were used and quantified in triplicates by duplex ddPCR assay. The lower LoD was determined as the lowest dilution which exhibited amplification in all reactions at that concentration. However, the LoQ was not only the lowest dilution which had 100% amplification, but also the lowest dilution which had consistent HIV DNA copy numbers per 10^6^ cells equivalents, as shown in the serially diluted Molts-IIIB DNA. We determined the threshold cutoff manually using 4 to 6 negative-control DNA, isolated from HIV-uninfected PBMCs and NTCs, in each assay. Theoretically, these controls should not emit fluorescence. Thus, thresholds were set above the detection of fluorescence for both negative controls (NCs) and NTCs in each experiment. Droplets that are located above threshold were considered positive droplets, while droplets placed below threshold were considered negative droplets. Inefficient PCR amplification which can be due to poor signal-to-noise separation causes the rain phenomenon. We optimized the poor signal-to-noise separation using dual quenched probes. In addition, false-positive droplets can be due to contamination of either the reagents or the lab environment. Thus, we aliquoted all reagents for one-time use and performed vigorous decontamination procedures for the lab environment. Finally, detection of a fluorescence above the thresholds was considered a positive PCR, whereas a fluorescence below the threshold was considered a negative PCR. QuantaSoft software automatically analyzed the positive droplets and converted it to copies/μL of target sequence.

### Total viral DNA assay by duplex ddPCR.

To develop and optimize the ddPCR assay, we analyzed Molt3-IIIB cells, a chronically infected cell line containing approximately 1 HIV provirus per cell and HIV-uninfected leukocyte, as a negative control. The ddPCR assay for measuring HIV-1 DNA was performed with a Bio-Rad QX 200 ddPCR system with automatic droplet generator. The published primers and probe sets used for this assay were the HIV-gag and RPP30 sequences (26). Total cell DNA was measured by ddPCR as RPP30 DNA copies/μL. Total HIV-1 DNA (detecting both integrated and unintegrated HIV-1 DNA) was measured using a forward primer in R of the HIV LTR (RU5-F), a reverse primer in U5 (RU5-R), and a FAM tagged probe in U5 modified with an internal ZEN quencher (RU5-Probe) (Table 3). The ddPCR reaction mix consisted of 2× ddPCR Supermix for probes (no dUTP), 500 nM primers, 250 nM probes for HIV-1 and RPP30, 10U restriction enzyme HaeIII, and DNA template. Droplet generation and transfer of emulsified samples to PCR plates was performed according to the manufacturer’s instructions (Auto QX200 Droplet Generator, Bio-Rad, Hercules, CA) followed by PCR with the following cycling conditions: 37°C for 60 min, 95°C for 10 min, 40 cycles of 95°C 30 s, and 58.1°C for 1 min. All reactions were tested in duplicate. NTC with nuclease-free water and DNA from noninfected leukocytes were included in every run as an NC. After PCR, the plate was loaded into the QX200 Droplet Reader, which analyzed each individual droplet and determined copy numbers using QuantaSoft software. These numbers were used to calculate HIV DNA copy per million cells. The RPP30 DNA copy was used as an internal control (reference gene) to normalize the data and report as copies/10^6^ cells equivalent.

**TABLE 3:** Sequences of the primers and probes used for ddPCR

Primer/probe ID	Sequence
Primer	
HIV1-RU5-FWD-ddPCR	5′-TA AGC CTC AAT AAA GCT TGC C-3′
HIV1-RU5-REV-ddPCR	5′-GTT CGG GCG CCA CTG CTA GA-3′
Alu-FWD-Integrated HIV PCR	5′-GCC TCC CAA AGT GCT GGG ATT ACA G-3′
RPP30-FWD-ddPCR	5′-AGA TTT GGA CCT GCG AGC G-3′
RPP30-REV-ddPCR	5′-GAG CGG CTG TCT CCA CAA GT-3′
Gag-REV-Integrated HIV PCR	5′-GTT CCT GCT ATG TCA CTT CC-3′
	
Probe	
HIV1-RU5-ddPCR ZEN	5′-/56-FAM/CC AGA GTC A/ZEN/C ACA ACA GAC GGG CAC A/3IABkFQ/-3′
RPP30-ddPCR ZEN	5′-/5HEX/TT CTG ACC T/ZEN/G AAG GCT CTG CGC G/3IABkFQ/-3′

### Integrated DNA assay development by nested ddPCR.

Alu-HIV PCR was performed as described earlier (14). This assay consists of two duplex assays: one to detect HIV-1, and one for a human gene, RPP30. The HIV-1 duplex assay combines information from two regions in the HIV-1 genome, which are the RU5 and gag regions. Alu-HIV PCR was performed by use of gag-reverse HIV primer and Alu-forward primer, as well as HIV-only PCR (gag reverse primer-only, Table 3). The DNA was diluted to 1 ng/μL or 5 ng/μL, and 25 μL of DNA was used in a 50-μL reaction. For the first PCR, 25 μL of master mix in a 50-μL PCR consisted of 1× GoTaq PCR mix, 1.25 U of GoTaq DNA polymerase, 200 nM ALU-FWD-Integrated HIV Primer, 1,200 nM Gag-REV-Integrated HIV primer, and 0.4 mM dNTP each. The Gag-REV primer-only reaction was run in parallel for each sample. The primer and probe sequences are shown in Table 3. DNA from noninfected leukocytes was used as a template for the negative-control reaction for background subtraction. The first PCR cycle was performed with the following conditions: 95°C for 2 min, and 40 cycles of 95°C for 15 s, 60°C for 15 s, and 72°C for 3.5 min. The product of the first PCR was used as the template for the nested ddPCR. The second PCR of the nested ddPCR and cycling conditions were the same as for the optimized HIV DNA-ddPCR conditions described above. All reactions were run in duplicate for the second PCR. After PCR, the plate was loaded into the QX200 Droplet Reader, which analyzed each individual droplet and determined copy numbers using QuantaSoft software. The data were normalized with the internal control, RPP30, followed by background normalization with data from gag-REV only reaction. The final data are reported as integrated HIV copies/10^6^ cells equivalent.

### Quantification of integrated HIV DNA by qPCR.

Integrated HIV DNA levels were quantified using two-step PCRs, as previously described (27, 28). Integrated HIV-1 DNA was pre-amplified with two Alu primers and a primer specific for the HIV-LTR region, in addition to primers specific for the CD3 gene to determine cell count. The products of the first round of amplification were used as the template for nested qPCR to amplify HIV and CD3 sequences. Copy number was determined by extrapolation against a 5-point standard curve (3 to 30,000 copies) performed in triplicate using extracted DNA from the ACH-2 cell line.

This article does not contain any studies with human participants performed by any of the authors. A Not Human Subjects Research determination was made by the Eastern Virginia Medical School Institutional Review Board.
