# Supplementary material for: Development of Droplet Digital PCR-Based Assays to Quantify HIV Proviral and Integrated DNA in Brain Tissues from Viremic Individuals with Encephalitis and Virally Suppressed Aviremic Individuals
Source: Microbiol Spectr. 2022 Jan 12;10(1):e00853-21. doi: 10.1128/spectrum.00853-21 (PMC8754137; doi:10.1128/spectrum.00853-21)
Supplement: SUPPLEMENTAL FILE 1 — Supplemental material. Download SPECTRUM00853-21_Supp_1_seq9.pdf, PDF file, 0.1 MB [file spectrum00853-21_supp_1_seq9.pdf]

Supplementary Table 1. Patient characteristics

ProjID	Category	PMI	Age at death	Sex	R	Risk	CD4	Plasma vl	CSF vl	Brain pathology
01640	Uninfected	19	63	M	H	-	-	-	-	normal
01923	Uninfected	6.5	61	M	H	-	-	-	-	Abn-Focal Infarct
00039	HIVE	8	46	M	W	Hom-sx	15	167,143	11,908	HIVE
00284	HIVE	4	36	M	H	Hom-sx	16	195,269	-	HIVE
00488	HIVE	5.5	42	M	W	Hom-sx	99	6,940	750,000	HIVE, MGNE
00918	HIVE	13	25	M	W	-	-	-	-	HIVE
01212	HIVE	5	37	M	W	Hom-sx	218	-	-	HIVE
01231	HIVE	6	46	M	B	Het-sx	178	15,001	116,413	HIVE
01555	HIVE	48	47	M	W	Hom-sx	9	210,000	-	HIVE
01580	HIVE	4	50	M	H	IVDU	1	58,419	-	HIVE
01598	HIVE	27	45	F	B	IVDU	6	-	-	HIVE
01994	HIVE	31	37	M	W	Hom-sx, IVDU	-	-	750,000	HIVE
02390	HIVE	17	63	F	B	Het-sx	25	730,085	-	HIVE
00006	Virally Suppressed	11.5	62	F	B	Het-sx	392	50	50	ischemic
00009	Virally Suppressed	11	50	F	W	Het-sx, IVDU	791	50	50	Abn-unspecified
00522	Virally Suppressed	24	66	M	W	Hom-sx	465	50	50	normal
00630	Virally Suppressed	18.5	61	M	B	Het-sx, IVDU	271	50	50	Abn-unspecified
00658	Virally Suppressed	6	52	M	W	Hom-sx	78	50	50	normal
00697	Virally Suppressed	21	66	M	W	Hom-sx	798	50	-	Abn-Focal Infarct
00699	Virally Suppressed	46.5	59	M	B	Hom-sx	491	50	-	ischemic
00719	Virally Suppressed	20	44	M	B	IVDU	219	50	50	ischemic
01374	Virally Suppressed	22.5	54	M	W	Hom-sx	491	85	50	Leukoencephalopathy
01495	Virally Suppressed	7	39	M	B	Het-sx	112	40	-	ischemic
01878	Virally Suppressed	8.5	63	M	W	Hom-sx	133	50	50	ischemic
01986	Virally Suppressed	8.5	69	M	W	Hom-sx	355	20	60	ischemic
02035	Virally Suppressed	18.23	50	M	H	Hom-sx	300	20	-	Abn-Alzheimer/Focal Infarct
02087	Virally Suppressed	14.5	51	M	W	Hom-sx	223	50	139	Abn-ChronicHTN
02104	Virally Suppressed	6	47	M	H	Hom-sx	61	48	-	Abn-Focal Infarct
02398	Virally Suppressed	4.5	64	M	B	Hom-sx	1,043	40	na	Abn-Focal Infarct

Supplementary Table 2a. Proviral (P) and integrated (I) HIV-1 DNA quantitated by ddPCR in the brain tissues of HIV-infected individuals

ProjID #	Group	Brain area<br>BG	 FWM	 CC
00039	HIVE	P: 3.19E+03<br>I: 3.96E+03	P: ND<br>I: ND	P: 6.80E+05<br>I: 5.16E+04
00284	HIVE	P: ND<br>I: ND	P: ND<br>I: ND	P: 5.68E+04<br>I: 5.43E+03
00488	HIVE		P: 1.89E+05	P: 1.66E+05<br>I: 3.44E+03
00918	HIVE	P: 3.17E+04<br>I: 8.94E+03	P: 4.42E+04	P: 4.05E+04<br>I: 1.60E+04
01212	HIVE	P: 1.66E+03<br>I: 5.49E+03	P: ND<br>I: ND	P: 8.56E+05<br>I: 9.19E+04
01231	HIVE		P: 1.23E+03<br>I: ND	
01555	HIVE	P: 8.85E+05	P: 1.29E+06	P: 2.66E+05
01580	HIVE	P: 8.82E+04<br>I: 1.66E+04	P: 1.94E+05<br>I: 4.39E+04	P: 4.41E+05<br>I: 6.43E+03
01598	HIVE	P: 9.77E+05<br>I: 1.77E+05	P: 2.90E+05<br>I: 5.03E+04	P: 6.05E+05<br>I: 8.61E+04
01994	HIVE	P: 7.73E+04<br>I: 1.90E+03	P: 5.23E+05	P: 3.68E+05<br>I: 4.30E+04
02390	HIVE	P: 1.09E+05<br>I: 6.39E+03	P: 2.58E+05<br>I: 1.45E+04	P: 3.08E+05<br>I: 1.57E+04
00006	Virally Suppressed	P: 1.17E+03<br>I: ND		P: 3.38E+01<br>I: ND
00009	Virally Suppressed	P: ND<br>I: ND	P: ND<br>I: ND	P: ND<br>I: ND
00522	Virally Suppressed	P: ND<br>I: ND	P: ND<br>I: ND	P: ND<br>I: ND
00630	Virally Suppressed	P: ND<br>I: ND	P: 1.38E+03	P: 1.28E+02<br>I: ND
00658	Virally Suppressed	P: 2.79E+02<br>I: 2.80E+02	P: ND<br>I: ND	P: ND<br>I: ND
00697	Virally Suppressed	P: ND<br>I: ND	P: ND<br>I: ND	P: ND<br>I: ND
00699	Virally Suppressed	P: ND<br>I: ND	P: ND<br>I: ND	P: 2.34E+01<br>I: ND
00719	Virally Suppressed	P: ND<br>I: ND	P: 2.70E+01<br>I: ND	P: ND<br>I: ND
01374	Virally Suppressed	P: ND<br>I: ND	P: 4.15E+00<br>I: ND	P: ND<br>I: ND
01495	Virally Suppressed	P: 1.28E+03<br>I: ND	P: 1.07E+03<br>I: ND	P: 7.13E+03<br>I: ND
01878	Virally Suppressed	P: ND<br>I: ND	P: ND<br>I: ND	P: ND<br>I: ND
01986	Virally Suppressed	P: ND<br>I: ND	P: ND<br>I: ND	P: ND<br>I: ND
02035	Virally Suppressed	P: 4.24E+01<br>I: ND	P: ND<br>I: ND	P: ND<br>I: ND
02087	Virally Suppressed	P: ND<br>I: ND		P: 1.07E+02<br>I: ND
02104	Virally Suppressed	P: ND<br>I: ND	P: ND<br>I: ND	P: ND<br>I: ND
02398	Virally Suppressed	P: ND<br>I: ND	P: ND<br>I: ND	P: 2.85E+01<br>I: ND
01640	Uninfected	P: ND		
01923	Uninfected	P: ND		

BG: basal ganglia, FWM: frontal white matter, CC: corpus callosum
ND, not detected
Cells greyed out indicate no tissues available.

Supplementary Table 2b. Proviral (P) and 2LTR (2) HIV-1 DNA quantitated by RT-qPCR

ProjID #	Group	Brain area<br>BG	 FWM	 CC
00284	HIVE	P: 2.58E+03<br>2: ND	P: 2.14E+05<br>2: 1.36E+03	
00918	HIVE		P: 1.66E+04<br>2: ND	
01212	HIVE	P: ND<br>2: ND	P: ND<br>2: ND	
01231	HIVE		P: ND<br>2: ND	
01555	HIVE	P: 1.12E+05<br>2: 3.58E+02	P: 4.58E+06<br>2: ND	P: 6.16E+05<br>2: 1.86E+02
01580	HIVE		P: 1.53E+05<br>2: 1.97E+03	
01598	HIVE			P: 4.00E+05<br>2: 2.20E+01
01994	HIVE			P: 7.02E+05<br>2: 1.06E+02
02390	HIVE		P: 3.97E+05<br>2: 1.45E+02	
00006	Virally Suppressed	P: ND<br>2: ND		P: ND<br>2: ND
00009	Virally Suppressed			P: ND<br>2: ND
00522	Virally Suppressed	P: ND<br>2: ND		
00630	Virally Suppressed	P: ND<br>2: ND		
00697	Virally Suppressed	P: ND<br>2: ND		
00699	Virally Suppressed	P: ND<br>2: ND		
00719	Virally Suppressed	P: ND<br>2: ND		
01495	Virally Suppressed			P: ND<br>2: ND
01878	Virally Suppressed	P: ND<br>2: ND		
02035	Virally Suppressed	P: ND<br>2: ND		
02087	Virally Suppressed			P: ND<br>2: ND
02398	Virally Suppressed			P: ND<br>2: ND

BG: basal ganglia, FWM: frontal white matter, CC: corpus callosum
ND, not detected
Cells greyed out indicate no tissues available.
